# Broken mirrors: a trainee’s experience of racism in the workplace

**DOI:** 10.3399/BJGPO.2020.0146

**Published:** 2020-11-25

**Authors:** Margaret Ikpoh

**Affiliations:** 1 GP Partner, Holderness Health, Hull, UK

**Keywords:** mirroring, racism, trainee, general practice, general practitioners

## Background

Racism involves a group who have the power to carry out systematic discrimination through the institutional policies and practices of the society and by shaping the cultural beliefs and values that support those racist policies and practices.^1^ A racist is one who supports those policies through their actions or interactions or expression of ideas.^2^ Racism in the NHS has been highlighted in both literature and by several institutions for several decades but there still exists a degree of denial that racism exists within the medical profession.^3^ Here, the experience of a trainee during a shared surgery is detailed — including the detrimental impact of the poor feedback he received — and suggestions are offered to tackle racism on GP training programmes.

### Introduction

Shared surgeries are a common method employed in practice that allows the GP trainer to observe their trainees, and guide them through their consultation while helping them to develop their consultation skills and gently correct them where necessary.^4^

Direct supervision with trainee and trainer working together and observing each other positively affects patient outcome and trainee development; constructive feedback is essential.^5^

Mirroring or limbic synchrony is a communication technique often used unconsciously during our communication with patients.^6^ It can be physical or verbal, and enables doctors — especially GP trainees — to establish better relationships and build rapport with patients.^7^

### A trainee’s experience

I recall a story from a Black male trainee, an excellent doctor who settled down well into the practice although he lacked a degree of self-confidence. He consulted patients often using the Calgary–Cambridge communication model, which had been taught to him as an undergraduate in communication modules delivered by a diverse communication faculty.^8^ He was also familiar with the technique of mirroring and demonstrated this subconsciously during assessments of his consultations. During a shared surgery, I had noticed a recurring theme with regards to his posture when consulting White patients, in that whether seated or standing, he always assumed a physical stance lower than that of the patient. It was rather, a subordinate posture almost akin to a servant bowing before their master.

Several months into his training, he finally admitted that in his previous practice, his White male trainer felt that his build (he is 6 feet tall) would be *’* *overwhelming and could instil fear in* *W* *hite patients* *’*. He was advised that he should try to avoid mirroring the patient in these circumstances and instead make himself physically smaller in the consultation. The trainee, keen to enhance his communication style, adopted this approach in surgery, in the hope that it would improve his consultation skills with White patients.

He declined to raise or discuss the incident with anyone previously, as he had found the episode stressful and embarrassing. This is consistent with our knowledge that doctors in training are reluctant to raise concerns for fear that it may jeopardise their career prospects.^9^

I was speechless upon hearing this and spent the rest of the session discussing the inappropriateness of the racist advice he was given, and the importance of maintaining one’s identity within the consultation. Once he left the tutorial, I cried because a trainer’s unconscious (or perhaps conscious) bias had stripped him of his dignity, both physically and mentally, and had a profound impact on his interpersonal skills within the consultation.

### Supportive environments

As trainers, we are influential and can have a positive impact on our trainees’ trajectories and self-belief. Although they are independent learners, research has shown that supportive learning environments influences learning.^10^

Suggestions for training schemes to ensure we provide such environments for our trainees include:

- The implementation of training on race equity for GP trainers;
- Increase awareness of cultural competence in the workplace;
- Consideration of active bystander training for trainers and trainees; and
- Offering support on the role of *Freedom to Speak Up Guardians* within primary care.

## Conclusion

I am often asked by various healthcare professionals about the need for ongoing storytelling when we already know racism exists. We all have stories that should not be forgotten, however having a space to relay our lived experiences and speak up or call out racism will form part of the action required to tackle it in the context of GP training.

If we are denied the freedom to express our traumas and to examine their root causes, as we do with any untoward event in practice, how can we ever hope to truly improve our status quo?

It is time for us to stand up tall and be counted.
